# Gender and Age Variations in Pharmacists’ Job Satisfaction in the United States

**DOI:** 10.3390/pharmacy7020046

**Published:** 2019-05-17

**Authors:** Manuel J. Carvajal, Ioana Popovici, Patrick C. Hardigan

**Affiliations:** 1 Department of Sociobehavioral and Administrative Pharmacy, College of Pharmacy, Nova Southeastern University, 3200 South University Drive, Fort Lauderdale, FL 33328-2018, USA; ip153@nova.edu; 2 College of Medicine, Nova Southeastern University, 3200 South University Drive, Fort Lauderdale, FL 33328-2018, USA; patrick@nova.edu

**Keywords:** age disparities, gender disparities, job satisfaction, job-related preferences, pharmacist workforce

## Abstract

While several studies have attested the presence of systematic gender and age variations in pharmacists’ satisfaction with their jobs, only a few of them have considered both classifications simultaneously. None have done so while systematically examining multiple facets of practitioners’ work. This article estimated U.S. pharmacists’ satisfaction levels with various facets of their work, compared them simultaneously between genders and among age groups, and tested for the presence of gender–age interaction effects. The study was based on self-reported survey data collected from 701 pharmacists (31.0% response rate). Mean and standard deviation values for 18 indices related to pharmacists’ work were calculated. When age groups were controlled, female pharmacists expressed overall higher levels of satisfaction with their job than male pharmacists; they also expressed greater satisfaction with multiple specific facets and with the profession, as well as greater workload and stress than male pharmacists. The findings revealed few significant differences among age groups and a limited gender–age interaction effect for pharmacists’ satisfaction with key facets of their work. These findings should contribute to the development and refinement of rational criteria for increasing sources of satisfaction in pharmacy settings.

## 1. Introduction

Organizational commitment issues (i.e., turnover, absenteeism, tardiness, theft, etc.) and quality of services rendered by pharmacists throughout the world have been linked to practitioners’ levels of satisfaction and dissatisfaction with various facets of their work [1,2,3,4,5,6,7,8,9,10,11,12,13,14,15]. While intuitively most analysts would agree that it is in the best interests of employers to keep their pharmacist employees happy, satisfaction with one’s work is an elusive concept pervaded by subjectivity and difficult to conceptualize and measure in an operational context. Not only is it interpreted differently by different persons that may experience a common set of conditions such as working in the same location or performing the same kind of work, but there are tradeoffs involved that often are perceived unequally. For example, the prospect of a higher paying job or a promotion may be a strong source of satisfaction for some practitioners, but the additional hours of work, stress, or possible relocation that the prospect entails may be even stronger sources of dissatisfaction for other practitioners. Further blurring the concept is a recent empirical finding of a weak association between pharmacists’ overall job satisfaction responses and reported satisfaction with several facets that were hypothesized to configure job satisfaction [16].

## 2. Background

The existence of systematic gender and age variations in how the facets of pharmacists’ jobs constitute sources of satisfaction or dissatisfaction, the tradeoffs among them, and their intensity is a commonly held platitude in the literature [17,18]. Work-related decisions usually are made within the context of household decision packages configured by the size and composition of the household, which are largely influenced by the gender-role ideology and age of the marital partners. Traditionally men and women experience socially defined, differentiated involvement with childcare and household responsibilities, with women picking up the brunt of the burden. Thus, compared to men, women are more likely to work part-time [19,20,21,22] and exhibit job-related preferences compatible with work-family balance such as hours of work, scheduling flexibility, and proximity of job site to home.

Work-related differences between men and women transcend family commitments and delve into intrinsic traits. Maier [23] argues that men primarily engage in competition to win and strive for success, subordinating other life activities to the pursuit of career goals, while women tend to search for equilibrium in life activities and are more interested than men in advancing others as well as themselves. He further argues that men use communication as a tool to solve problems, establish status, and convey independence while women use communication largely to establish connections. If these views are correct, one would expect male practitioners to be more motivated than their female counterparts by the adequacy of salaries and benefits, autonomy, and availability of advancement opportunities facets of their work, and female practitioners to be more motivated than their male peers by facets such as fairness in the workplace, job atmosphere, and relations with coworkers.

Age differences in pharmacists’ satisfaction and dissatisfaction with various facets of their work also have been reported across the continents [1,4,5,10,11,15,24,25,26,27,28,29,30,31]. Invariably these studies have found that younger pharmacists are more dissatisfied with their job, or some part of it, than older pharmacists. Younger practitioners possess little workforce experience and may not be able to assess their working conditions accurately; the gap between expectations and reality may contribute to their dissatisfaction. As they grow older, their aspirations are reduced and they realize that they face limited choices in the workplace [32,33] and are likely to attach less importance to professional ambitions, acquiring more awareness of areas within the profession from which they derive greater levels of satisfaction.

Younger versus older worker comparisons have a deeper perspective that goes beyond mere age differences. This perspective focuses on the role that work plays in practitioners’ lives. Baby boomers, who comprise the older segment of the pharmacist workforce, commonly are portrayed as a generation characterized by solid work ethics, with clearly defined professional goals, a drive for material success, and commitment to their employers. They frequently consider work as the most important part of their lives, and define themselves in terms of what they do. Conversely, pharmacists younger than baby boomers exhibit a preference for autonomy and flexible work schedules, express more interest in family and close friends than in material success, are motivated by the latest technologies, and view organizations with cynicism and contempt [34]. Younger pharmacists criticize baby boomers for being too competitive, overly cautious, and loyal to their organizations beyond reason, while baby boomers view younger pharmacists’ emphasis on balancing work and leisure as lack of commitment and an erosion of work ethics. In short, their values and expectations are different, which probably translates into different sources of satisfaction and dissatisfaction with the various facets of their work.

Independently of their respective trends and influence on the configuration of job satisfaction patterns, gender and age may have some interaction effect. For example, younger women are prone to experience more work-family conflicts than other gender-age group combinations [35] for several reasons. Besides being in the family formation years, which makes them more susceptible to children’s time intensive demands, their jobs are frequently viewed by themselves and others as a secondary income source, with needs and preferences of their own that may not coincide with the needs and preferences of older women or younger men. Their participation in the labor force tends to be countercyclical; when many men become unemployed or are forced to work fewer hours during a downturn of the business cycle, younger women tend to work more to compensate for the loss of household income [36,37]. Another asymmetry is found in the distribution of costs and benefits of family migration; younger women are less likely than their husbands to initiate moves, or resist moves initiated by their husbands, because their gains/losses from migration frequently are surpassed by their husbands’ losses/gains [38].

At the other end of the spectrum, older workers continue to work more years than their predecessors partly because of their relatively better health and longevity. The trend away from defined benefit pension plans and toward defined contribution plans, plus the ability to receive full social security benefits beyond a certain age regardless of labor force participation, provide incentives to remain active in the workforce [39,40], oftentimes on a part-time basis [41]. Older male pharmacists experience issues that may be of little or no concern to younger male practitioners or older female practitioners, so their perceived sources of satisfaction or dissatisfaction at work may be different from those of other practitioners of the same gender and/or the same age group. Their motivating factors to seek part-time employment include, among others, avoiding depletion of savings, supplementing retirement benefits, fighting boredom, and validating their personal worth [42].

## 3. Objectives

Within the context of the background outlined in the previous section, this article sought to (1) estimate U.S. pharmacists’ levels of satisfaction with various facets of their work, (2) compare simultaneously levels of satisfaction between genders and among age groups, and (3) test for the presence of a gender–age interaction effect. While many studies have addressed the influence of both gender and age on the configuration of pharmacists’ job satisfaction patterns, only a few of them have taken into account both classifications at the same time and none has done so in examining systematically multiple facets of practitioners’ work. It is important to analyze both classifications simultaneously in order to avoid attributing variation in satisfaction to one classification that should be attributed to the other classification or to the interaction of both. The study is important because a uniform set of job-related rewards and incentives (or disincentives) offered by employers to practitioners of both genders and all age groups may not be adequate if indeed significant differences are observed.

## 4. Methods

This study was based on self-reported survey data. Participants were asked to assess their satisfaction with key facets of their work as pharmacists using 0-to-10 intensity scales, with 10 denoting the greatest intensity. This procedure posed the advantage of response homogeneity, as practitioners were able to rate their experience with various facets of their work using a common measurement standard, with more room for discrimination in their responses than is normally provided by the traditional Likert scale. The rather narrow range of options ensured the adequacy of the mean as a measure of central tendency since there was no room for outliers. The indicator used here has been applied successfully in previous studies.

### 4.1. Indices

Eighteen assessment indices related to pharmacists’ work were included in the analysis. Most of these had to do with important aspects of their job, but three were not job-related: assessment of satisfaction with the profession, own capability as a pharmacist, and own performance as a pharmacist. The other indices focused on adequacy of earnings and benefits, importance of earnings and benefits, amount of workload, stress, job security, availability of advancement opportunities, autonomy, scheduling flexibility, supervisor’s support, relations with coworkers, fairness in the workplace, job atmosphere, importance of job to patients, importance of job to the organization, and job satisfaction in general.

Mean and standard deviation values for each index were calculated by gender and age group. The analysis was conducted for each gender-age group cell as well as for both genders within each age group and all age groups within each gender. The disaggregation by gender and age group took into consideration different values previously reported in the literature for male and female pharmacists [43,44] as well as for practitioners of different ages [34,45]. The disaggregation for both of these classifications is not commonly found for any other independent variable. As a general rule and consistently with earlier work [16], overall average index values under 3.00 were considered low, values ranging from 3.00 to 6.99 were considered moderate, and values 7.00 and above were considered high. The cutoff point for statistical significance was *p* = 0.10. In addition, mean and standard deviation values were calculated by gender and age group for the wages and salaries reported by the pharmacists in the sample to compare actual earnings with the adequacy perceived by respondents in the different categories.

### 4.2. Statistical Model

A two-way classification model with multiple replications was designed to probe the nature of differences in indices related to job satisfaction. One classification consisted of both genders (*i* = 1, 2). The other classification identified three age groups (*j* = 1, ..., 3): under 45 years old, 45–59 years old, and 60 years or older. Within each gender-age group cell, n_*ij*_ replications were observed. This design posed the advantage of allowing not only gender and age-group differences to be tested simultaneously and independently of each other, but also testing a gender–age group interaction effect. The model has been applied successfully in the analysis of variations in pharmacists’ earnings and other variables [17].

The linear additive model for each index was as follows:

X_*ijk*_ = μ + γ_*i*_ + α_*j*_ + (γα)_*ij*_ + ε_*ijk*_ (1)

where

X_*ijk*_ was the index value reported by the *k*th pharmacist in the *j*th age group and the *i*th gender;
μ was the overall mean;
γ_*i*_ was the systematic effect of the *i*th gender;
α_*j*_ was the systematic effect of the *j*th age group;
(γα)_*ij*_ was the gender-age group interaction effect;
ε_*ijk*_ was the stochastic disturbance (random error) term of the *k*th pharmacist in the *j*th age group and the *i*th gender;

and where

*i* = 1 for men and *i* = 2 for women;
*j* = 1 for pharmacists under 45 years of age, *j* = 2 for 45–59 year-old pharmacists, and *j* = 3 for pharmacists 60 years or older; and
n_*ij*_ was the number of pharmacists of the *i*th gender and the *j*th age group reporting their index value for each index assessing satisfaction with key facets of their work.

### 4.3. Data

The survey data were gathered from responses to a questionnaire sent by the authors to 2400 pharmacists practicing throughout the United States. These pharmacists were selected by Medical Marketing Services (MMS) using a simple random scheme. MMS is a leading provider of lists of pharmacists and other healthcare professionals. Its data depository includes pharmacists from all fields within the profession; approximately 90% of the estimated 281,560 U.S. registered pharmacists in 2012 [46] were included in the MMS data file.

The survey questionnaire, previously validated [16,17], was exclusively designed for this and other pharmacist workforce studies. It was mailed in March 2012 and a reminder was sent two weeks later. No major environmental or other factors affecting job satisfaction were anticipated since 2012. The sample size was chosen according to Cochran’s formula developed for categorical and other outcomes [47], with a 5% sampling error. The research effort was supported solely by internal university funds, and institutional review board approval was secured to conduct the probe.

## 5. Results

Of the 2400 questionnaires mailed to potential participants, 139 packets were returned undelivered for various reasons. A total of 701 pharmacists participated in the study by providing answers to all relevant questions. The number of observations and the response rate (31.0%) compared favorably with those reported by similar undertakings [2,48,49,50,51]. Out of the total respondents, 403 pharmacists (57.5%) were men and 298 pharmacists (42.5%) were women; in terms of age, 27.0% were less than 45 years old, 45.6% were 45–59 years old, and 27.4% were 60 years or older. Reflecting the “womanization” of the profession and the demographics of the population from which they were drawn, male pharmacists were outnumbered by female pharmacists under 45 years of age, while the opposite occurred with older pharmacists.

### 5.1. Indices

The means and standard deviations of the 18 assessment indicators reported by pharmacists are presented in Table 1. All aggregate values, with the exception of perceived importance of job to patients, were in the moderate range; perceived importance of job to patients was slightly higher. Importance of job to patients, own capability as a pharmacist, and amount of workload were reported as the top three aggregate index values; availability of advancement opportunities, perceived support from one’s supervisor, and scheduling flexibility were ranked, in that order, at the bottom of the scale.

The average index values of female pharmacists were consistently greater than the values reported by their male counterparts, and the differences were statistically significant except for adequacy of earnings and benefits, availability of advancement opportunities, autonomy, fairness in the workplace, and job atmosphere. Significant differences among age groups were detected only for the amount of workload and perceived job importance to patients, and the gender–age group interaction effect was significant only for perceived adequacy of earnings and benefits and perceived job importance to the organization.

### 5.2. Wages and Salaries

The empirical evidence showed significant differences in wages and salaries between genders (see Table 2). On average, male pharmacists in the sample earned 11.2% higher wages and salaries than their female counterparts for an estimated gap of 10.1%. This gender gap varied by age group: 10.8% for pharmacists under 45 years old, 19.0% for pharmacists 45–59 years old, and –2.0% for pharmacists 60 years or older; in other words, female older pharmacists earned, on average, 2.0% higher wages and salaries than their male peers. Yet the interaction effect was not statistically significant, nor were differences among age groups.

## 6. Discussion

Several findings are worth noting in this analysis of U.S. pharmacists’ assessment of satisfaction with key facets of their work. The first is that when age groups were controlled, female pharmacists expressed overall higher levels of satisfaction with their job than male pharmacists; they also expressed greater satisfaction with multiple specific facets as well as with the profession. These findings accorded with those of other studies that did not account for variation in age [6,16,52,53]. Women’s relatively greater overall job and professional satisfaction levels were observed in every age group, but the differences were wider for younger than older practitioners.

Women also reported consistently greater satisfaction than men in every age group when assessing the quality of their supervisor’s support, relations with coworkers, and perceived importance of job to patients. These findings were in line with those of previous studies regarding women’s perceived importance of supervision issues [54,55,56], interaction with fellow workers [57], and the nature of tasks performed [15,53]. In four other job facets in which gender differences were statistically significant—importance of earnings and benefits, job security, scheduling flexibility, and importance of job to the organization—female pharmacists’ estimated response values were greater than the values of male pharmacists, but women 60 years or older actually registered lower values than the estimated values reported by men 60 years or older. Yet the gender–age group interaction effect was not statistically significant for any of the four indices.

Despite the relatively greater satisfaction experienced by female pharmacists regarding different facets of their work, they reported higher levels of workload and stress than male pharmacists, and the inequalities were observed at all ages. Usually an excessive workload constitutes a major source of dissatisfaction for pharmacists [1,2,6,10,14,15,51,58]. The incongruence was particularly confounding in light of the finding of a previous study whereby female practitioners actually worked fewer hours than male practitioners [16].

Another observed incongruence was the absence of a gender differential in perceived adequacy of salaries and benefits, especially when men in the sample earned 11.2% higher wages and salaries than women. This incongruence has been documented in the literature as the paradox of the contented female worker [54,59,60,61]. Several plausible explanations for this paradox have been postulated [18]. One is that women feel less pressure to exceed at work than men because of the traditional division of labor; men are primarily responsible for the household’s financial well-being and women are primarily responsible for housework and childcare. Another plausible explanation may be that women have lower labor outcomes expectations than men, so their goals are more easily fulfilled. A third explanation suggests that additional earnings contribute more to the job satisfaction of men than to women’s job satisfaction [62,63], which leads women to compensate for the foregone satisfaction of less pay with social aspects such as interaction with patients, good supervisors, congenial coworkers, and scheduling flexibility, all of which registered gender satisfaction differentials.

In any event, perceived adequacy of salaries and benefits exhibited a statistically significant gender–age group interaction effect: relatively more women than men under 60 years of age thought that their salaries and benefits were adequate, but the opposite was the case for practitioners 60 years or older. Although the interaction effect of reported wages and salaries lacked statistical significance, female practitioners under 60 years of age reported lower earnings than male pharmacists of the same age groups, and the opposite was true for practitioners 60 years or older. Thus, the evidence observed here points toward an even greater incongruence that may be more adequately termed “the paradox of the contented male and female pharmacists.”

In general, pharmacists in the sample thought highly of their professional capability and performance. In both instances women reported higher scores than men, and the differences were significant. However, also in both instances, men 60 years or older reported higher satisfaction scores than women in the same age group. Perhaps further research may shed some light into why the satisfaction relationship between the genders is atypical for the 60 years or older group compared to younger practitioners.

Another important finding in this paper was the paucity of significant differences among age groups or an interaction effect when pharmacists assessed their satisfaction with key facets of their work. This paucity was contrary to commonly accepted platitudes found in the literature [18]. In the only two indices that were statistically significant for age-group differences, pharmacists 45–59 years old reported more dissatisfaction with their workload and gave higher ratings to the importance of their jobs to patients than did younger or older pharmacists. Notwithstanding the absence of statistically significant differences among age groups, a common pattern of women scoring higher values in the less than 60 years age group, but lower values among older pharmacists, in seven indices should not be ignored; it might not have occurred by chance alone.

## 7. Limitations

Job satisfaction is a subjective issue. Not only is it subject to different interpretations by different people, even when they share a common background and professional outlook, but its assessment by any one person may fluctuate over time, depending on the person’s emotions and feelings. It is virtually impossible to fully standardize as a variable. This is the first constraint that limits the interpretation of the findings presented in this paper. A second limitation is that the study rested on a cross-sectional survey, so there was no measurement of whether or how practitioners’ satisfaction with key facets of their work varied with time or was influenced by changing labor market conditions affecting the pharmacist workforce or the ongoing revamping of the healthcare system. Self-reported data were used, which opened the door to validity and reliability criticism even though the questionnaire was tested prior to being mailed to participants.

The study was limited to analyzing the influence of gender and age group on reported satisfaction. Other factors such as marital status, number of children, or the amount of outstanding student loans, not considered here, may affect pharmacists’ satisfaction with key facets of their work independently of gender or age, may be gender or age selective, or may contribute to the interaction effect. This limitation may be addressed in a subsequent study.

Another limitation is that although the MMS data files utilized in configuring the sample were broad-based and included about 90% of pharmacists practicing in 2012, the sample might not have been representative of the other 10%. Furthermore, there was no way to ensure that the questionnaires were received or completed by the intended respondents, which was another limitation. In addition, some analysts might criticize the 31.0% response rate as relatively low. Information about earnings and satisfaction with different facets of one’s work is considered by some people as of an intimate nature, so some practitioners might have been reluctant to share it. No incentives such as monetary remuneration or a chance to win a raffle prize were used to motivate participation, which might have altered the number of respondents. In any event, the sample consisted of 701 observations, a sizable data set whose volume compensated for a modest response rate.

## 8. Conclusions

Despite its limitations, this study was successful in estimating U.S. pharmacists’ levels of satisfaction with various facets of their work, comparing simultaneously their levels of satisfaction between genders and among age groups, and testing for the presence of a gender–age interaction effect. It identified the facets in which practitioners scored highest and lowest satisfaction levels, and found that when age was controlled, female pharmacists were consistently more satisfied with most facets of their work than male pharmacists. Since men and women responded differently to multiple facets, a uniform set of job-related rewards and incentives (or disincentives) offered by employers to practitioners of both genders may not be adequate.

The seemingly atypical behavior exhibited by pharmacists 60 years or older (compared to younger pharmacists), whereby men’s levels of satisfaction with several facets of their work were greater than those of their female counterparts, should be analyzed more thoroughly. A potentially significant trend along these lines, discovered in future research, might signal a need for further adjustment by employers in their efforts to keep their pharmacist employees happy and manage a diverse workforce more effectively. Perhaps the age-group boundaries in this study were not specified properly, and practitioners with heterogeneous needs and preferences were placed together, thereby weakening the statistical outcomes of the age-group classification and the gender-age interaction effect. The specification of different age groups might yield different results.

Finally, it is important to observe that while pharmacists’ satisfaction with their work remains an elusive concept plagued with subjectivity, this study was successful in conceptualizing and applying a methodological framework that produced concrete results. The empirical evidence obtained here, and the conclusions derived from it, should be regarded as preliminary in nature, but, hopefully, they will contribute to the development and refinement of rational criteria for increasing sources of satisfaction in pharmacy settings.

## Figures and Tables

**Table 1:** Means and standard deviations (in parentheses) of indices related to pharmacists’ work by gender and age group.

Index and Statistical Significance	Age Group	Gender		
		Men (*i* = 1)	Women (*i* = 2)	Both Genders
Number of observations	Under 45 years old (*j* = 1)	71 (n_11_)	118 (n_21_)	189
	45–59 years old (*j* = 2)	173 (n_12_)	147 (n_22_)	320
	60 years or older (*j* = 3)	159 (n_13_)	33 (n_21_)	192
	All age groups	403	298	701
Adequacy of earnings and benefits Interaction effect (*p* = 0.038)	Under 45 years old (*j* = 1)	5.45 (3.12)	6.93 (2.60)	6.38 (2.88)
	45–59 years old (*j* = 2)	5.64 (3.00)	6.37 (2.70)	5.97 (2.88)
	60 years or older (*j* = 3)	7.27 (2.63)	6.29 (1.96)	7.08 (2.53)
	All age groups	6.21 (2.99)	6.58 (2.58)	6.37 (2.82)
Importance of earnings and benefits Between genders (*p* = 0.038)	Under 45 years old (*j* = 1)	6.03 (3.02)	7.11 (2.65)	6.70 (2.83)
	45–59 years old (*j* = 2)	5.73 (3.00)	7.17 (2.37)	6.37 (2.82)
	60 years or older (*j* = 3)	6.90 (2.83)	6.59 (2.37)	6.84 (2.74)
	All age groups	6.21 (2.99)	7.08 (2.47)	6.58 (2.80)
Amount of workload Between genders (*p* = 0.015)	Under 45 years old (*j* = 1)	6.01 (3.27)	7.03 (2.55)	6.65 (2.88)
	45–59 years old (*j* = 2)	6.83 (2.51)	7.39 (2.06)	7.09 (2.33)
	60 years or older (*j* = 3)	6.75 (2.37)	6.81 (2.25)	6.76 (2.34)
	All age groups	5.86 (3.30)	7.18 (2.29)	6.88 (2.50)
Stress Between genders (*p* ≤ 0.001)	Under 45 years old (*j* = 1)	5.86 (3.30)	6.96 (2.56)	6.54 (2.90)
	45–59 years old (*j* = 2)	6.54 (2.74)	6.95 (2.50)	6.73 (2.64)
	60 years or older (*j* = 3)	5.78 (3.14)	6.81 (2.62)	5.95 (3.08)
	All age groups	6.12 (3.02)	6.94 (2.53)	6.47 (2.85)
Job security Between genders (*p* = 0.043)	Under 45 years old (*j* = 1)	5.54 (2.99)	6.84 (2.44)	6.34 (2.73)
	45–59 years old (*j* = 2)	5.70 (3.11)	6.13 (2.81)	5.90 (2.98)
	60 years or older (*j* = 3)	6.09 (3.30)	5.97 (2.86)	6.07 (3.22)
	All age groups	5.82 (3.16)	6.39 (2.69)	6.06 (2.98)
Availability of advancement opportunities	Under 45 years old (*j* = 1)	3.42 (2.68)	4.11 (3.15)	3.85 (2.99)
	45–59 years old (*j* = 2)	3.34 (2.86)	3.37 (3.03)	3.35 (2.93)
	60 years or older (*j* = 3)	3.10 (3.20)	3.61 (2.90)	3.19 (3.15)
	All age groups	3.26 (2.97)	3.69 (3.07)	3.44 (3.02)
Autonomy	Under 45 years old (*j* = 1)	5.55 (2.78)	6.27 (2.37)	5.99 (2.55)
	45–59 years old (*j* = 2)	5.80 (2.70)	6.14 (2.86)	5.96 (2.78)
	60 years or older (*j* = 3)	5.93 (2.96)	5.59 (2.01)	5.87 (2.82)
	All age groups	5.81 (2.82)	6.13 (2.60)	5.94 (2.73)
Scheduling flexibility Between genders (*p* = 0.045)	Under 45 years old (*j* = 1)	5.11 (3.02)	6.15 (3.15)	5.76 (3.14)
	45–59 years old (*j* = 2)	5.27 (3.22)	6.27 (3.09)	5.72 (3.20)
	60 years or older (*j* = 3)	5.88 (3.27)	5.55 (2.86)	5.82 (3.20)
	All age groups	5.48 (3.21)	6.14 (3.09)	5.76 (3.18)
Supervisor’s support Between genders (*p* = 0.005)	Under 45 years old (*j* = 1)	5.07 (2.88)	6.00 (3.04)	5.65 (3.01)
	45–59 years old (*j* = 2)	5.00 (3.25)	5.92 (3.32)	5.42 (3.31)
	60 years or older (*j* = 3)	5.51 (3.62)	6.15 (2.68)	5.62 (3.48)
	All age groups	5.21 (3.34)	5.98 (3.13)	5.54 (3.27)
Relations with coworkers Between genders (*p* = 0.009)	Under 45 years old (*j* = 1)	6.01 (3.08)	7.27 (2.24)	6.79 (2.65)
	45–59 years old (*j* = 2)	6.25 (2.90)	6.86 (2.56)	6.53 (2.76)
	60 years or older (*j* = 3)	6.63 (2.92)	6.70 (2.67)	6.64 (2.87)
	All age groups	6.36 (2.94)	7.00 (2.45)	6.63 (2.76)
Fairness in the workplace	Under 45 years old (*j* = 1)	6.13 (2.73)	6.38 (2.51)	6.29 (2.59)
	45–59 years old (*j* = 2)	5.78 (2.88)	6.08 (2.88)	5.92 (2.88)
	60 years or older (*j* = 3)	6.18 (3.36)	5.94 (2.68)	6.14 (3.25)
	All age groups	6.00 (3.05)	6.19 (2.71)	6.08 (2.91)
Job atmosphere	Under 45 years old (*j* = 1)	5.92 (2.48)	6.53 (2.59)	6.30 (2.56)
	45–59 years old (*j* = 2)	5.70 (2.95)	6.39 (2.72)	6.02 (2.86)
	60 years or older (*j* = 3)	6.42 (3.07)	6.18 (2.40)	6.38 (2.96)
	All age groups	6.02 (2.93)	6.42 (2.63)	6.19 (2.81)
Importance of job to patients Between genders (*p* = 0.001) Among age groups (*p* = 0.063)	Under 45 years old (*j* = 1)	5.70 (3.71)	7.42 (2.64)	6.77 (3.18)
	45–59 years old (*j* = 2)	6.15 (3.49)	7.87 (2.54)	6.91 (3.21)
	60 years or older (*j* = 3)	7.62 (2.86)	7.94 (0.97)	7.68 (2.60)
	All age groups	6.62 (3.38)	7.70 (2.45)	7.08 (3.06)
Importance of job to the organization Between genders (*p* = 0.016) Interaction effect (*p* = 0.002)	Under 45 years old (*j* = 1)	5.15 (3.21)	7.64 (2.12)	6.70 (2.84)
	45–59 years old (*j* = 2)	5.99 (3.22)	7.18 (2.67)	6.51 (3.04)
	60 years or older (*j* = 3)	7.56 (2.81)	6.53 (2.72)	7.36 (2.80)
	All age groups	6.42 (3.19)	7.28 (2.48)	6.78 (2.94)
Overall job satisfaction Between genders (*p* = 0.024)	Under 45 years old (*j* = 1)	5.76 (2.91)	6.45 (2.48)	6.19 (2.66)
	45–59 years old (*j* = 2)	5.60 (3.19)	6.60 (2.72)	6.06 (3.02)
	60 years or older (*j* = 3)	6.14 (3.28)	6.24 (2.50)	6.16 (3.15)
	All age groups	5.84 (3.18)	6.50 (2.60)	6.12 (2.96)
Professional satisfaction Between genders (*p* ≤ 0.001)	Under 45 years old (*j* = 1)	5.73 (2.69)	7.19 (1.96)	6.64 (2.37)
	45–59 years old (*j* = 2)	5.81 (3.00)	6.95 (2.76)	6.33 (2.94)
	60 years or older (*j* = 3)	6.28 (3.19)	6.67 (2.34)	6.35 (3.06)
	All age groups	5.98 (3.03)	7.01 (2.42)	6.42 (2.83)
Own capability as a pharmacist Between genders (*p* = 0.093)	Under 45 years old (*j* = 1)	6.18 (3.46)	7.31 (3.19)	6.89 (3.32)
	45–59 years old (*j* = 2)	6.22 (3.49)	7.72 (2.70)	6.88 (3.24)
	60 years or older (*j* = 3)	7.37 (2.96)	6.76 (2.80)	7.25 (2.93)
	All age groups	6.64 (3.33)	7.45 (2.91)	6.98 (3.18)
Own performance as a pharmacist Between genders (*p* = 0.018)	Under 45 years old (*j* = 1)	5.91 (3.80)	7.35 (2.96)	6.81 (3.36)
	45–59 years old (*j* = 2)	5.80 (3.75)	7.71 (3.01)	6.65 (3.56)
	60 years or older (*j* = 3)	7.23 (3.33)	6.94 (2.79)	7.17 (3.22)
	All age groups	6.35 (3.65)	7.48 (2.96)	6.83 (3.42)

**Table 2:** Estimated means and standard deviations (in parentheses) of pharmacists’ wages and salaries by gender and age group.

Age Group	Gender		
	Men	Women	Both Genders
Under 45 years old	121,463	108,395	113,388
	(31,896)	(31,135)	(31,978)
45–59 years old	126,350	102,330	115,055
	(108,660)	(33,390)	(83,087)
60 years or older	104,798	106,914	105,143
	(67,700)	(20,238)	(62,432)
All age groups	116,951	105,177	111,923
	(84,131)	(31,437)	(67,126)

Differences between genders are statistically significant (*p* = 0.061).
